# Impaired adhesion of induced pluripotent stem cell-derived cardiac progenitor cells (iPSC-CPCs) to isolated extracellular matrix from failing hearts

**DOI:** 10.1016/j.heliyon.2018.e00870

**Published:** 2018-10-18

**Authors:** Elizabeth N. McKown, Joshua L. DeAguero, Benjamin D. Canan, Ahmet Kilic, Yiliang Zhu, Paul M.L. Janssen, Dawn A. Delfín

**Affiliations:** a The University of New Mexico College of Pharmacy, Department of Pharmaceutical Sciences, MSC09 5360, 1 University of New Mexico, Albuquerque, NM 87131, USA; b The Ohio State University College of Medicine, Department of Physiology and Cell Biology and the Davis Heart Lung Research Institute, 200 Hamilton Hall, 1645 Neil Avenue, Columbus, OH 43210, USA; c The Ohio State University College of Medicine, Department of Surgery and the Davis Heart Lung Research Institute, Richard M. Ross Heart Hospital, 452 West 10th Ave., Columbus, OH 43210, USA; d The University of New Mexico School of Medicine, Department of Internal Medicine, MSC10 5550, 1 University of New Mexico, Albuquerque, NM 87131, USA

**Keywords:** Cell biology, Stem cell research, Cardiology, Pathology

## Abstract

We tested the hypothesis that induced pluripotent stem cell-derived cardiac progenitor cells (iPSC-CPCs) are less able to adhere to the extracellular matrix (ECM) derived from failing human hearts with dilated cardiomyopathy compared to nonfailing human heart ECM. We also hypothesized that morphological development, cell beating rates, and mRNA levels of Nkx2.5 and cardiac troponin T would be altered after culturing the iPSC-CPCs on the failing heart ECM under cardiomyocyte differentiation conditions. We used microscopy to distinguish between adhered and unadhered cells, and to determine morphological development and cell beating. We used qPCR to determine mRNA levels. iPSC-CPCs show a significantly reduced ability to adhere to the ECM of failing hearts and higher expression of Nkx2.5 mRNA. However, morphological development, cell beating rates, and cardiac troponin T levels were not significantly altered in the cells cultured on the failing heart ECM. Our study shows that the failing heart ECM from patients with dilated cardiomyopathy impairs initial iPSC-CPC adhesion and may have a modest effect on the ability of the cells to transdifferentiate into cardiomyocytes.

## 1 Introduction

The extracellular matrix (ECM) of the human heart is a complex microenvironment of proteins, glycoproteins, proteoglycans, glycosaminoglycans and basement membrane proteins (Rienks et al., 2014), which, along with cells, comprise the mammalian myocardium. Under healthy conditions, the heart's ECM is a scaffold that anchors cells, provides organ structure, and supports contraction mechanics (Beachley et al., 2015; Frangogiannis, 2017; Rienks et al., 2014; Segura et al., 2014; Yabluchanskiy et al., 2013; Yang et al., 2015). Due to its importance to organ function, the ECM is maintained under strict homeostatic balance (Segura et al., 2014; Yabluchanskiy et al., 2013). Various cardiovascular diseases put undue stress on the heart, causing it to undergo pathological remodeling, which may alter the ECM (Fan et al., 2012; Kong et al., 2014). Although this remodeling is initially compensatory, unchecked it becomes decompensatory, reducing cardiac function over time, and potentially resulting in heart failure (Segura et al., 2014).

In heart failure with dilated cardiomyopathy, the left ventricular volume increases alongside thinning of the ventricular wall. Pathological remodeling of the myocardium in dilated cardiomyopathy is characterized by wide-spread interstitial fibrosis (expansion of the ECM in between cardiomyocytes) (Sheppard, 2011). The pathologically remodeled ECM has altered biochemical and biophysical properties compared to healthy tissue (Fan et al., 2012; Kong et al., 2014; Rienks et al., 2014; Segura et al., 2014; Yabluchanskiy et al., 2013). We have shown that the global protein content in the ECM of failing human hearts with dilated cardiomyopathy (DCM-failing) is significantly changed (DeAguero et al., 2017), while others have documented changes in specific proteins (Marijianowski et al., 1995). Alterations in the remodeled ECM are associated with detrimental effects on the cells that adhere to or reside within it (Rienks et al., 2014).

Presently, advanced, end-stage pathological cardiac remodeling and heart failure can only be cured by a heart transplant, since the heart has minimal regenerative capacity (Bergmann et al., 2009; Piek et al., 2016). The limitations of heart transplantation, such as insufficient availability of donor hearts and organ rejection, necessitate the discovery of novel treatments for end-stage disease (Leong et al., 2017). Stem cell therapy is an exciting prospective treatment option under active preclinical and clinical investigation (Duelen and Sampaolesi, 2017; Lader et al., 2017; Nigro et al., 2018; Sanganalmath and Bolli, 2013). These stem cells are expected to integrate into the myocardium and differentiate into cardiomyocytes that function in concordance with healthy, existing cardiomyocytes, or differentiate into other cardiac cells types as required for repair of the damaged tissue. The disappointing reality of cardiac stem cell therapy clinically, is that more than 98% of the cells are not detectable in the myocardium post-transplantation and the benefit to clinical outcomes is inconsistent at best (Aicher et al., 2003; Brenner et al., 2004; Terrovitis et al., 2008, 2006). A number of theories have been put forward to explain the massive loss of cells, including that the stem cells may simply not adhere to the pathologically remodeled myocardium. Cells, including transplanted stem cells, anchor to the ECM in the myocardium through focal adhesions. Lack of ECM attachment can lead to anoikis (cell death due to non-adhesion to the ECM), and can make the cells susceptible to clearance by blood flow. Biochemical and biophysical properties of the ECM itself can profoundly affect the phenotype of stem cells (Beachley et al., 2015; Taylor et al., 2017). Moreover, the ECM of pathologically remodeled hearts is especially hostile toward cell adhesion and other biochemical aspects of cell survival (Byron et al., 2013).

The reduced-adhesion theory of pathologically remodeled hearts has not yet been rigorously tested for cardiac stem cells. Therefore, our goal was to test the hypothesis that cardiac stem cells are less able to adhere to the altered extracellular matrix of DCM-failing hearts compared to nonfailing hearts. Additionally, we assessed whether cells that adhered to the ECM of DCM-failing hearts would show altered capacity for cardiomyocyte transdifferentiation. To conduct our study, we decellularized human heart left ventricular tissue, leaving behind isolated ECM. We coated tissue culture plates with this human heart ECM, then assessed initial cell adhesion to the DCM-failing versus nonfailing cardiac ECM. We continued to culture the cells under cardiomyocyte differentiation conditions. Under these conditions, we also assessed morphological changes to the cells over several days, cell beating rates, and markers of cardiomyocyte transdifferentiation. The data we now present show that there are functional differences between DCM-failing and nonfailing ECM in terms of their effects on stem cell phenotypes. We hope that continued studies on the interaction between the human heart ECM and cardiac stem cells can lead to translating ECM alterations, especially with regard to protein expression, into possible therapeutic targets to improve cardiac stem cell therapy success.

## 2 Materials and methods

### 2.1 Human heart samples

The analysis of human heart tissue in this study was performed under the guidelines of the Declaration of Helsinki and was approved by the Institutional Review Boards at The Ohio State University (protocol no. 2012H0197) and the University of New Mexico (protocol no. 13-358). Nonfailing human heart tissues were procured from organ donors who expired from causes other than heart failure, who were not diagnosed with heart failure prior to death, but whose hearts were deemed unsuitable for organ transplant. Failing human heart tissues were procured as explants from patients with end-stage heart failure receiving a transplant. Details of clinical and histological data related to the nonfailing and failing heart tissues used in this study can be found in (DeAguero et al., 2017). We focused on the three nonfailing and DCM-failing human hearts that were used to generate the proteomics data in (DeAguero et al., 2017).

### 2.2 Tissue decellularization and ECM isolation

Flash-frozen human heart tissue was thawed and finely minced. Minced tissue, weighing 2–5 g, from each individual sample was added to decellularization wash (10 ml per 2–5 g tissue; 2.5 mM ethylenediaminetetraacetic acid [EDTA], 1x protease inhibitor cocktail [Sigma S8820], 100 U penicillin,10 μg/ml streptomycin [Sigma P0781], and 50 μg/ml gentamycin [Sigma G1272] in deionized, distilled water). After a 30-min incubation on a rotator, the decellularization wash was removed and replaced with 10 ml of decellularization buffer (addition of 1% sodium dodecyl sulfate to decellularization wash). After a 6 h incubation on the rotator, the spent decellularization buffer was removed and replaced with fresh buffer. For 5–7 days at 8–12 h intervals, the spent decellularization buffer was replaced with fresh buffer. When the tissue appeared white and translucent, decellularization was complete. Decellularization wash was added to the tissues and incubated on the rotator. After the 24 h incubation, the decellularization wash was removed and replaced with fresh decellularization wash for 3 × 5-min washes. Decellularized tissue was flash frozen and lyophilized. Complete decellularization was confirmed by Masson's trichrome staining of ECM tissue sections (DeAguero et al., 2017).

### 2.3 Isolated ECM for cell culture coatings

Lyophilized ECM was cryogenically milled in a sterile environment until the ECM was ground into a fine powder. ECM was suspended in phosphate-buffered saline (PBS) and protease inhibitor cocktail (Sigma S8820) at 15 mg/ml, then filtered through a stainless-steel mesh strainer. Protein concentrations were quantified using a Qubit 2.0 Fluorimeter (Life Technologies/Thermo Fisher Scientific Q32866) and Qubit Protein Assay Kit (Thermo Fisher Scientific Q33212). Using non-tissue culture treated multi-well plates, ECM suspension at concentration of 5 μg protein/cm^2^, was placed within the appropriate wells, then incubated overnight at 37 °C. The wells were rinsed with PBS containing 50 μg/ml gentamycin (Sigma G1272) and amphotericin B (Sigma A2942) and allowed to dry. As a positive control, wells were coated with fibronectin (Sigma F1141) solution in PBS at 5 μg/cm^2^. No-coating (negative control) wells were simply left uncoated.

### 2.4 iPSC-CPC assay

**Fig. 1:**
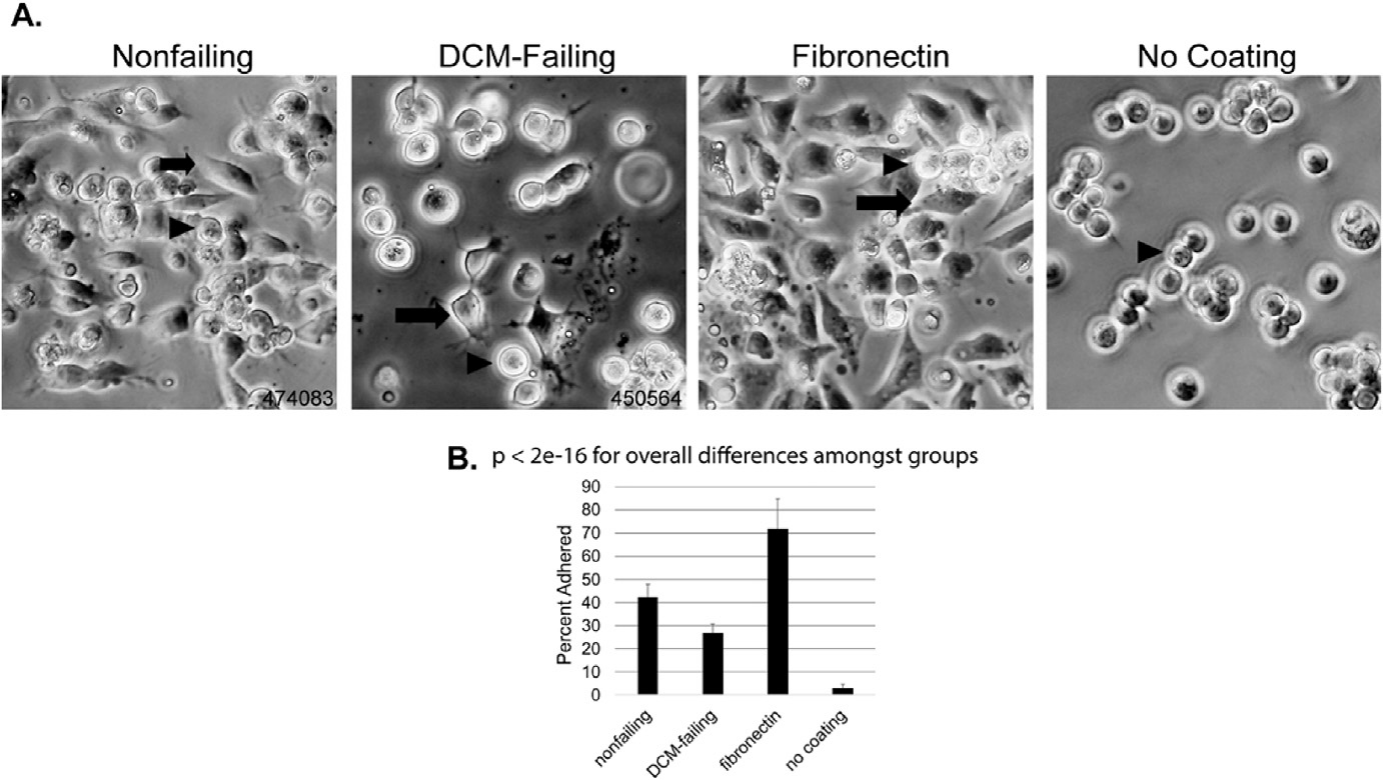
iPSC-CPC adhesion to the extracellular matrix from human hearts. Cardiac progenitor cells were allowed 1 hour to adhere to the extracellular matrix derived from nonfailing human hearts (n = 3), DCM-failing human hearts (n = 3), fibronectin (positive control) or no coating on tissue culture plasticware (negative control). DIC images were acquired and analyzed by Olympus CellSens software for adhered cells (oblong with low refractivity, arrows) or unadhered cells (spherical with high refractivity, arrowheads). Representative images from four replicates of the assay are shown. Sample numbers for the human heart ECMs depicted are provided. B) The average percentage of adhered cells out of the total cell count in a defined area (250 mm^2^) was determined over four separate experiments. Error bars show the standard error of the data. All pairwise differences were highly statistically significant (p < 1 × 10^−4^ by Tukey's Honest Significant Difference).

We plated 1.6 × 10^5^ human induced pluripotent stem cell-derived cardiac progenitor cells (iPSC-CPCs, Cellular Dynamics, Inc.) per cm^2^ in the tissue culture plates after following the supplier's instructions for thawing and handling the cells. We then applied differentiation medium (Williams E medium [Gibco A12176], 4% cell maintenance cocktail [Gibco A13448], 50 μg/ml gentamycin [Sigma G1272], XAV939 10 μM [Sigma X3004], 2.5 μM SB431542 [Sigma 431542], 0.2% dimethylsulfoxide [DMSO, solvent for stock solutions of XAV939 and SB431542], and 1 mM Y-27632 (Tocris Biosceinces 1254]), to the cells for 24 hours. We allowed the cells to adhere for 1 hour, then took differential interference contrast (DIC) images on an Olympus IX83 microscope. We performed four separate replicates of the experiment. We analyzed cellular sphericity and refraction using Olympus CellSens Count and Measure software (version 1.13) to analyze adhesion. The person doing the analysis was masked to the sample identity, and image analysis software was used to assess sphericity, to maximize analytical objectivity. More spherical and highly refractive cells were considered unadhered, whereas adhered cells were ellipsoid or beginning to become spindle shaped from attached podophilia, and lost refractivity (Fig. 1). Without changing the medium or removing the cells that did not adhere within the first 24 hours of the experiment, we then cultured the cells with differentiation medium without Y-27632 between 24-48 hours of culture. Finally, we cultured the cells with maintenance medium (Williams E medium [Gibco A12176], 4% cell maintenance cocktail [Gibco A13448], 50 μg/ml gentamycin [Sigma G1272]) for another 4 days. The total cell culture time was 6 days. Once per day, we took DIC images to assess morphological changes over time. After 6 days, one day after the cells began to beat, we took DIC videos for 60 or more seconds to assess average beats per minute. At the end of 6 days, we harvested the cells for further analysis. These longer-term cell culture assays were performed in three replicates.

### 2.5 RNA isolation and qPCR

At the end of 6 days in culture, the medium was removed and 500 μl chilled TRIzol (Invitrogen Cat #15596018) was added to the cell culture wells. The plate was stored at -20 °C. The cells were lysed by vigorous pipetting then a QIAShredder kit (Qiagen 79654) was used for homogenization, following the TRIzol supplier's procedure for RNA isolation, followed by Qiagen RNeasy Mini Kit (Qiagen 74104) for RNA cleanup. The concentration of RNA was measured with a Qubit RNA HS Assay Kit (Thermofisher Scientific Q32855) on a Qubit 2.0 spectrophotometer (Thermofisher Scientific Q32866). cDNA amplification was carried out using ThermoFisher's High Capacity cDNA Reverse Transcription Kit (ThermoFisher 4368814) and protocol at 100 ng RNA per 20 μl reaction. The kit and protocol from PowerSYBR Green PCR Master Mix (Applied Biosystems/Life Technologies 4367659) was used for quantitative real-time PCR on a ViiA-7 Real-Time PCR System (ThermoFisher 4453536). The qPCR was performed in two separate technical replicates for each of the three assay replicates. The primers were supplied by RealTimePrimers.com, using the following sequences (forward/reverse):

Human Beta-2-Microglobulin (*B2M*): 5′-TGCTGTCTCCATGTTTGATGTATCT-3′/5′-TCTCTGCTCCCCACCTCTAAGT-3′
Human TATA Binding Protein (*TBP*): 5′-TATAATCCCAAGCGGTTTGC-3′/5′-GCTGGAAAACCCAACTTCTG-3′
Human NK2 transcription factor related, locus 5 (*NKX2-5*): 5′-CCCTGGATTTTGCATTCACT-3′/5′-GGGGACAGCTAAGACACCAG-3′
Human Troponin T Type 2 (*TNNT2*): 5′-GACCTGCAGGAGAAGTTCAA-3′/5′-GACGAGCAGATCTTTGGTGA-3′
Human Ki67 (*KI67*): 5′-ATCCAAAAGGATTCCCTCAG-3′/5′-AGGCGTATTAGGAGGCAAGT-3′

### 2.6 Statistics

Statistical differences in cell adhesion, gene expression by qPCR, and beats per minute were determined by linear mixed-effects models. Random effects were used in the models to capture within-sample-replication clustering and between-sample-replication variation. Wilcoxon rank sum test and t-test were used to verify results consistency. Tukey's Honest Significant Difference (HSD) and False Discovery Rate (FDR) were considered to adjust for multiple comparisons. Differences were considered statistically significant if *p* < 0.05.

## 3 Results and discussion

### 3.1 iPSC-cardiac progenitor cells adhere less to the DCM-failing heart extracellular matrix

The ECM from DCM-failing and nonfailing human hearts was decellularized and reconstituted into a suspension. The suspension was then put into tissue culture plates. As a positive control, fibronectin was plated at the same concentration. Other wells were left uncoated as negative controls. iPSC-CPCs were then cultured in the prepared plates. To count the cells at 1 hour post-plating in a 250 mm^2^ defined area in the center of the well, we used DIC microscopy imaging and image analysis software (Olympus CellSens), differentiating between adhered cells (non-spherical with reduced refractivity) and unadhered cells (spherical with high refractivity). We observed that significantly fewer cells were adhered to the ECM derived from DCM-failing hearts compared to nonfailing hearts (Fig. 1). Approximately 60% as many iPSC-CPCs adhered rapidly to the DCM-failing ECM, and about one-third as many when comparing DCM-failing ECM-exposed cells to fibronectin-cultured cells. Although a straightforward assay, the data were highly reproducible over four experiments and the positive control (fibronectin) showed the greatest adhesive ability while the negative control (no coating) show the least. These data show that the DCM-failing heart ECM does not support robust stem cell adhesion relative to the nonfailing heart ECM within 1 hour.

In the clinical setting, cardiac stem cells would likely have very limited time in the myocardium to adequately adhere to the ECM, and our data suggest that the DCM-failing ECM worsens the opportunity for adhesion relative to nonfailing ECM. This result supports that there is a lack of rapid integration of the stem cells into the myocardium before they are subject to being carried away by blood flow or succumbing to anoikis, resulting in the massive loss of stem cells over a short period of time that has been observed clinically (Aicher et al., 2003; Brenner et al., 2004; Terrovitis et al., 2008, 2006). This highlights the need to discover novel opportunities to increase rapid stem cell-ECM interaction and binding. Many bio-engineering groups are exploring cardiac stem cell patches, supported by a synthetic or naturally-derived matrix, that may adequately increase contacts between stem cells and the heart's ECM (Chiu et al., 2012; Ye et al., 2013).

Alternatively, or perhaps concurrently, we could use information on the specific protein alterations in the ECM of DCM-failing hearts to discover novel proteins to upregulate or downregulate in the heart, to force the stem cells to anchor to the ECM tightly and rapidly. In our proteomics study assessing the protein profile of the ECM derived from DCM-failing hearts compared to nonfailing hearts, we found that 12 of the 14 ECM-specific proteins were downregulated in DCM-failing hearts, including several that could be implicated in affecting cell-ECM adhesion. The ECM proteins that were present at lower levels in DCM-failing hearts were: 40S ribosomal protein SA, collagen IV α2, collagen IV α6, collagen XV α1, ECM protein 1, fibulin, integrin β-1 binding protein, inter-α-trypsin inhibitor heavy chain H1, proteoglycan 3, target of Nesh-SH3, tenascin, and von Willebrand factor A domain containing protein 1 (DeAguero et al., 2017). Our lab is currently investigating one of these promising candidates for the purpose of increasing iPSC-CPC adhesion to the ECM of failing hearts.

It is important to consider that, while our studies were performed on relatively-uniformly pathologically remodeled, failing human hearts with dilated cardiomyopathy, a substantial proportion of the patients who might receive cardiac stem cell therapy would be patients with heterogenous remodeling patterns, such as those who suffered from a myocardial infarction. It is notable, then, that cardiac stem cells may adhere less to damaged tissue, where they are most needed, than more healthy tissue in these patients. This possibility needs to be rigorously tested.

### 3.2 Morphological development and cell beating rates of iPSC-cardiac progenitor cells are similar during culturing on DCM-failing and nonfailing ECM

**Fig. 2:**
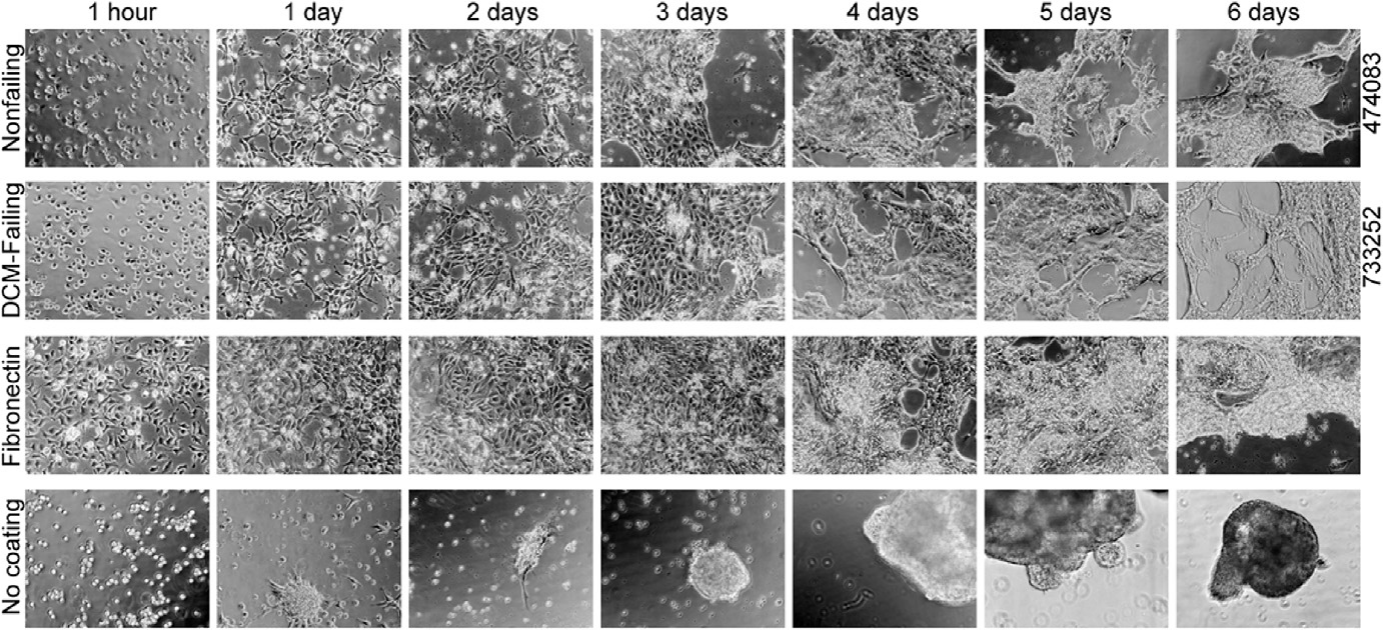
Morphological transdifferentiation of iPSC-CPCs cultured on the ECM from human hearts. iPSC-CPCs were cultured on the ECM derived from nonfailing human hearts (n = 3), DCM-failing human hearts (n = 3), fibronectin (positive control) or no coating on tissue culture plasticware (negative control). The images from three separate experiments were analyzed for differences in morphological development over 6 days. Representative images are shown. Sample numbers for the human heart ECMs depicted are provided.

We qualitatively assessed the morphological changes of the cells over 6 days of culture using DIC microscopy. We did not observe major differences between cells cultured on DCM-failing ECM versus nonfailing ECM (Fig. 2). The analysis revealed that cells on fibronectin appeared to proliferate faster and were marginally more confluent and cells on non-coated wells were sparse and very clumpy. At day 5, the cell sheets began to beat regularly. At day 6, we assessed the beats per minute by taking DIC videos of the cells then counting the number of beats over a defined time period (Table 1). We found that the beats per minute were highly variable from experiment to experiment despite consistency in culture and analysis techniques. This variability led to no discernable differences in beating rate between any of the samples.

**Table 1:** **iPSC-CPC beating rates after cells were cultured on the ECM from human hearts.** Average beats per minute were determined after cells were cultured on nonfailing heart ECM (n = 3), DCM-failing ECM (n = 3), fibronectin, or wells with no coating over three separate experiments. A linear mixed effects model revealed no statistically significant differences between the groups. Tukey's Honest Significant Difference is reported here.

Growth substrate	BPM (±SD)	*p*-values	
Nonfailing heart ECM	15.3 (±16.4)	vs. failing fibronectin no coating	=0.95 =0.82 =0.79
DCM-failing heart ECM	13.1 (±14.5)	vs. fibronectin no coating	=0.96 =0.94
Fibronectin	10.0 (±6.5)	vs. no coating	=1.00
No coating	9.7 (±6.3)	–	

Key: BPM = Beats per minute, SD = standard deviation.

Our study is underpowered to make strong conclusions about the differences in morphological or physiological transdifferentiation. However, the trends support that once adhered, the cells take a similar path of morphological transdifferentiation on any adhesive substrate, whether human heart ECM or fibronectin, including time to beating. Additionally, cell beating may be either similar regardless of the growth substrate or our assay does not have sufficient sensitivity to test the differences at 6 days post-culturing. The possible clinical implication if there is no actual difference in the cells' morphological development nor their ability to beat is that once adhered, the iPSC-CPCs will behave similarly upon exposure to the pathologically remodeled heart tissue as it would in a healthy heart. Thus, the remodeled ECM would not be an impediment to (nor stimulus of) these two important aspects of iPSC-CPC transdifferentiation. The persistent challenge in promoting iPSC-CPC transdifferentiation into cells with similar morphology and function to mature cardiomyocytes remains elusive (Karakikes et al., 2015; Lundy et al., 2013), but our data support that the pathologically remodeled cardiac ECM may not have a specific, significant effect.

### 3.3 Molecular markers of cardiomyocyte differentiation are variable in iPSC-CPCs cultured on DCM-failing and nonfailing heart ECM

A marker of cardiac stem cell lineage, the transcription factor Nkx2.5 (gene *NKX2-5*), is expressed in the iPSC-CPCs. As the cells transdifferentiate toward mature cardiomyocytes, their expression of the contractile protein cardiac troponin T (cTnT, gene *TNNT2*) is expected to increase, especially in proportion to Nkx2.5 (Forough et al., 2011). Additionally, as cardiac stem cells differentiate into mature cardiomyocytes, their proliferation levels decrease, and thus levels of proliferation markers, such as Ki67 (gene *KI67*), decrease correspondingly. We determined Nkx2.5, cardiac troponin T, and Ki67 mRNA levels by qPCR.

In the qPCR analysis, the threshold count (C_T_) values of the genes of interest were normalized to the housekeeping gene *B2M* to calculate the ΔC_T_ values (C_T_ of gene of interest subtracted by the C_T_ of housekeeping genes). The average ΔC_T_s using *B2M* are shown in Table 2. We verified the data using *TBP* as the housekeeping gene (not shown), which followed similar trends.

**Table 2:** **Average *NKX2-5*, *TNNT2*, and *KI67* mRNA levels, normalized to the housekeeping gene *B2M*, of iPSC-CPCs cultured on different growth substrates**. Average ΔC_T_ values were determined after cells were cultured on nonfailing heart ECM (*n* = 3), DCM-failing ECM (*n* = 3), fibronectin, or wells with no coating over three separate experiments and two technical replicates per experiment, subtracting the *B2M* C_T_ values from those of the gene of interest. A mixed effects model, in which variations were characterized as nested random effects, was used to determine statistically significant differences between the groups.

Growth substrate	ΔC_T_ value (±SD)	Comparisons	*p*-values	Fold-difference
***NKX2-5***				
Nonfailing heart ECM	0.39 (±1.17)	vs. failing fibronectin no coating	=0.03 <0.0001 =0.02	=0.78 =5.78 =1.58
DCM-failing heart ECM	0.04 (±1.09)	vs. fibronectin no coating	<0.0001 =0.002	=7.26 =2.01
Fibronectin	2.90 (±3.12)	vs. no coating	<0.03	=0.28
No coating	1.05 (±1.16)	–	–	–
***TNNT2***				
Nonfailing heart ECM	−2.45 (±1.13)	vs. failing fibronectin no coating	=0.06 <0.0001 =0.01	=0.80 =5.17 =2.01
DCM-failing heart ECM	−2.78 (±0.75)	vs. fibronectin no coating	<0.0001 =0.0003	=6.50 =2.53
Fibronectin	−0.08 (±2.49)	vs. no coating	=0.12	=0.39
No coating	−1.44 (±1.56)	–	–	–
***KI67***				
Nonfailing heart ECM	11.72 (±2.38)	vs. failing fibronectin no coating	=0.37 =0.19 =0.15	=0.78 =0.57 =1.87
DCM-failing heart ECM	11.38 (±2.41)	vs. fibronectin no coating	=0.24 =0.005	=0.73 =2.36
Fibronectin	10.92 (±1.09)	vs. no coating	<0.0001	=3.25
No coating	12.62 (±1.48)	–	–	–

Key: ΔC_T_ = normalized threshold cycle, SD = standard deviation.

*NKX2-5* mRNA ΔC_T_ values were 10-fold higher in the iPSC-CPCs cultured on the DCM-failing heart ECM compared to those on the nonfailing heart ECM. While this difference was determined to be statistically significant, it only represented a 1.3-fold higher gene expression level in the cells from the DCM-failing heart ECM using the ΔΔC_T_ method (Livak and Schmittgen, 2001). This difference is modest compared to the 7.3-fold higher expression in cells from DCM-failing heart ECM versus fibronectin, for example. Cells cultured on both nonfailing and DCM-failing heart ECM showed higher *NKX2-5* mRNA than cells on either fibronectin or no coating. These experiments do not clarify whether the alterations in *NKX2-5* expression are due to global alterations in all iPSC-CPCs, or alterations in a subset of cells. A future experiment to definitively show the pattern of alteration can be to perform Nkx2.5 protein immunofluorescence staining on the cells grown on the different matrices, and determine the pattern of localization or delocalization from the cells.

*TNNT2* mRNA ΔC_T_ values between iPSC-CPCs on nonfailing versus DCM-failing were not significantly different, although the trend showed a modestly higher expression in the DCM-failing iPSC-CPCs. Cells cultured on fibronectin had the lowest levels of *TNNT2* mRNA while those on either human heart ECM had the highest levels, and those on no coating had intermediate levels. We compared the ratio of *TNNT2* expression (absolute value) to *NKX2-5* expression, where higher numbers would correlate with higher expression of the mature cardiomyocyte marker (*TNNT2*) relative to the immature marker (*NKX2-5*), thus indicating more advanced transdifferentiation toward mature cardiomyocytes. From this analysis, the least cell maturation was observed in cells cultured on fibronectin (0.03), followed by those on no-coating (1.37). The human heart ECM seemed to promote maturation. Cells cultured the DCM-failing heart ECM showed the most maturation (69.50), while those on the nonfailing heart ECM (6.28) had a value on the same order of cells on no-coating. Although no differences in cell morphological development nor beating rates were observed microscopically, cells cultured on the DCM-failing heart ECM express greater tendency toward maturity at the gene expression level. Additional genetic markers to assess cell transdifferentiation toward mature cardiomyocytes will need to be tested to verify this finding, especially considering the differences in TNNT2 expression between cells cultured on nonfailing and DCM-failing heart ECM were not actually significantly different.

Differences in cell maturation gene expression in iPSC-CPCs cultured on pathologically remodeled tissue, compared to normal tissue, may impact functioning of the cells clinically. For example, infarcted hearts have regions of extensive pathological remodeling at the locus of the infarct, relatively healthy tissue remote from the infarct, and still different pathology in the infarct border zone. Cells that integrate into disparate regions of this heterogeneous heart may display different molecular phenotypes, which may result in disparate functioning and may promote arrhythmias.

With regard to cell proliferation, we observed that *KI67* mRNA levels were similar between cells cultured on the nonfailing and DCM-failing heart ECM, and both were similar to fibronectin. Therefore, the proliferation rates between cells on any matrix are not different. Cells cultured on no coating had significantly lower expression of *KI67* mRNA than cells on DCM-failing ECM and fibronectin, indicating that the proliferation of cells cultured on no coating was less than cells cultured on a matrix base. Clinically, iPSC-CPCs may not proliferate differently depending on whether the matrix is pathologically remodeled or not.

## 4 Conclusion

We have shown that the human heart ECM derived from patients with heart failure and DCM, which has undergone widespread interstitial fibrosis, has a negative influence of initial iPSC-CPC adhesion. However, once the cells have adhered, on a microscopic level, they appear to follow a similar path of transdifferentiation toward cardiomyocytes. On a molecular level, transdifferentiation may not be equivalent when cells are exposed to the pathologically remodeled ECM compared to normal ECM. The clinical consequence of this observation is that in hearts with heterogeneous remodeling, such as ischemic or infarcted hearts, transplanted stem cells may display different functional phenotypes that could lead to arrhythmias or other negative outcomes.

Our study was limited in scope; thus, our findings will need to be verified with a larger-scale study. Due to the extremely limited resource of human hearts available for our *in vitro* study, we analyzed only 3 hearts per group. The hearts we studied have corresponding proteomics data showing the change in protein expression in the DCM-failing hearts compared to the nonfailing hearts (DeAguero et al., 2017). Data in which significant differences between groups were observed, such as cell adhesion ability, we believe hold strong potential to be scientifically relevant, and should be studied further toward improving stem cell treatment of heart failure. Even data in which the differences between groups were not significant may still hold scientific relevance. A larger scale study with additional samples can reveal which aspects are important to consider in stem cell therapy.

We chose to focus on iPSC-CPCs as our cardiac stem cell model. However, many sources of stem cells are being assessed in pre-clinical and clinical research for heart failure treatments. It is possible that other types of stem cells would have a different response to the human heart ECM than the iPSC-CPCs we used in this study. Additional research should be conducted to understand the response of other types of stem cells to the failing heart ECM.

Still, we believe that we can glean important principles from our study. Stemming from the data we presented, we will research novel ways to promote cell adhesion to the pathologically remodeled ECM from human hearts, as well as novel ways to promote cell differentiation toward mature cardiomyocytes, with a particular focus on proteins that are altered in the DCM-failing hearts compared to nonfailing hearts. In all, we expect that this work will support that, as hypothesized, cardiac stem cells show a reduced ability to adhere to the pathologically remodeled ECM.

## Declarations

### Author contribution statement

Elizabeth McKown, Joshua DeAguero, Dawn Delfin: Conceived and designed the experiments; Performed the experiments; Analyzed and interpreted the data, wrote the paper.

Benjamin Canan, Paul Janssen: Performed the experiments, Contributed reagents; materials, analysis tools or data.

Ahmet Kilic: Contributed reagents, materials, analysis tools or data.

Yiliang Zhu: Analyzed and interpreted the data; Contributed reagents, materials, analysis tools or data.

### Funding statement

This work was supported by the American Heart Association (grant numbers 15BGIA22840012, 18AIREA33900010), the CTSC KL2 program (NIH grant number UL1TR001448), the National Human Genome Research Institute/FlyBase (grant number R25HG007630), and the University of New Mexico College of Pharmacy, and Cardiovascular and Metabolic Disease Signature Program.

### Competing interest statement

The authors declare no conflict of interest.

### Additional information

No additional information is available for this paper.
